# Head motion in the UK Biobank imaging subsample: longitudinal stability, associations with psychological and physical health, and risk of incomplete data

**DOI:** 10.1093/braincomms/fcae220

**Published:** 2024-07-02

**Authors:** Joey Ward, Simon R Cox, Terry Quinn, Laura M Lyall, Rona J Strawbridge, Emma Russell, Jill P Pell, William Stewart, Breda Cullen, Heather Whalley, Donald M Lyall

**Affiliations:** School of Health and Wellbeing, University of Glasgow, G12 8TB, Glasgow, UK; School of Philosophy, Psychology and Language Sciences, University of Edinburgh, EH8 9JZ, Edinburgh, UK; School of Cardiovascular and Metabolic Sciences, University of Glasgow, G12 8TA, Glasgow, UK; School of Health and Wellbeing, University of Glasgow, G12 8TB, Glasgow, UK; School of Health and Wellbeing, University of Glasgow, G12 8TB, Glasgow, UK; Cardiovascular Medicine Unit, Department of Medicine Solna, Karolinska Institute, 171 64, Stockholm, Sweden; Health Data Research (HDR)-UK, NW1 2BE, London, UK; School of Psychology and Neuroscience, University of Glasgow, G12 8QB, Glasgow, UK; School of Health and Wellbeing, University of Glasgow, G12 8TB, Glasgow, UK; School of Psychology and Neuroscience, University of Glasgow, G12 8QB, Glasgow, UK; Department of Neuropathology, Queen Elizabeth University Hospital, G51 4TF, Glasgow, UK; School of Health and Wellbeing, University of Glasgow, G12 8TB, Glasgow, UK; Centre for Clinical Brain Sciences, University of Edinburgh, EH16 4SB, Edinburgh, UK; School of Health and Wellbeing, University of Glasgow, G12 8TB, Glasgow, UK

**Keywords:** imaging, motion, bias, epidemiology, UK Biobank

## Abstract

Participant motion in brain magnetic resonance imaging is associated with processing problems including potentially non-useable/incomplete data. This has implications for representativeness in research. Few large studies have investigated predictors of increased motion in the first instance. We exploratively tested for association between multiple psychological and physical health traits with concurrent motion during T_1_ structural, diffusion, average resting-state and task functional magnetic resonance imaging in *N* = 52 951 UK Biobank imaging subsample participants. These traits included history of cardiometabolic, inflammatory, neurological and psychiatric conditions, as well as concurrent cognitive test scores and anthropometric traits. We tested for stability in motion in participants with longitudinal imaging data (*n* = 5305, average 2.64 years later). All functional and T_1_ structural motion variables were significantly intercorrelated (Pearson *r* range 0.3–0.8, all *P* < 0.001). Diffusion motion variables showed weaker correlations around *r* = 0.1. Most physical and psychological phenotypes showed significant association with at least one measure of increased motion including specifically in participants with complete useable data (highest β = 0.66 for diabetes versus resting-state functional magnetic resonance imaging motion). Poorer values in most health traits predicted lower odds of complete imaging data, with the largest association for history of traumatic brain injury (odds ratio = 0.720, 95% confidence interval = 0.562 to 0.923, *P* = 0.009). Worse psychological and physical health are consistent predictors of increased average functional and structural motion during brain imaging and associated with lower odds of complete data. Average motion levels were largely consistent across modalities and longitudinally in participants with repeat data. Together, these findings have implications for representativeness and bias in imaging studies of generally healthy population samples.

## Teaser

In ∼53 000 participants from the UK Biobank imaging subsample, Ward *et al*. show significant associations between poorer psychological/physical health, with increased head motion during structural and functional magnetic resonance imaging. People with poorer psychological and physical health were also significantly less likely to have complete imaging data.

## Graphical Abstract

**Graphical Abstract:**
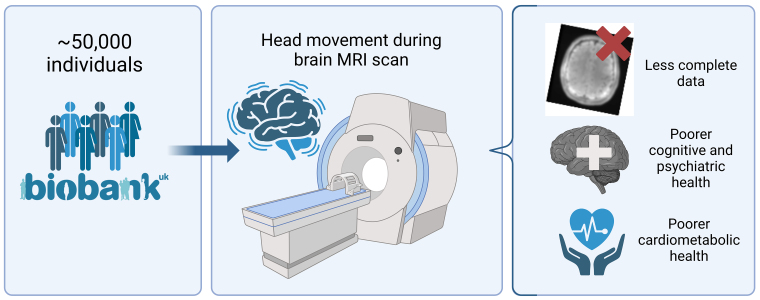

## Introduction

Structural and functional magnetic resonance imaging [(f)MRI] are important methods in understanding the human brain. These approaches include understanding underpinnings of neuropsychiatric, neurodegenerative and fundamental psychological processes,^1,2^ e.g. phenotype versus structural/functional brain variation and case–control designs. However, limitations and confounds to such an approach include volunteer bias^3^ and aspects of intra-scan behaviour. Motion during scanning including in generally healthy people (among other issues) can lead to artefact(s), shading and blurring of images.^4,5^ This can result in variable scan quality and non-useable data.^6^ For decades, MRI research has aimed for diagnostic and risk prediction utility with regard neuropsychiatric and neurodegenerative disorders. This literature can be impacted if confounding variables (e.g. head motion and artefacts) vary non-randomly, particularly in reports with relatively small sample sizes. It is therefore important to (as definitively as possible) understand predictors of increased head motion in a relatively large sample of generally healthy volunteers.

During an MRI scan, participants are instructed to remain as still as possible. While there are known health conditions that can make this difficult, even in generally healthy samples, motion occurs through sudden involuntary movements (e.g. sneezing), peripheral nerve stimulation, conscious movements due to discomfort or unintentional movements, e.g. tremors.^7^ Relatively small movements can lead to substantial contamination of data—instruction, training and restraints as they currently stand are not necessarily sufficient preventions.^8^ Methods for post-scan correction of motion exist, and these approaches have respective strengths and weaknesses, but can be very effective.^9^

The quality of imaging data may influence study conclusions and effect estimates, even after statistical correction for motion.^10^ Rueter *et al*.,^4^ for example, showed an accuracy loss of roughly 0.7% on cortical grey matter measurement per millimetre/per minute of average subject motion, in repeated imaging (in *N* = 12). There is a potential role for motion in irreproducible results, such that removal of scans with more motion can attenuate some associations.^6^ Roalf *et al*.^11^ demonstrated in participants that the inclusion of ‘poor’ quality data (in terms of artefacts) significantly reduced the correlation between increasing age and metrics of white matter diffusion [fractional anisotropy (FA) and mean diffusivity (MD); *N* = 374] leading to an underestimation of effect. Using *n* = 41 985 participants from UK Biobank, Alfaro-Almagro *et al*.^12^ showed that head motion (based on 48 variables), among other factors such as scanner table position, to varying extents confounded associations between imaging-derived phenotypes (IDPs) with cognitive and anthropometric variables.

Having established that individual differences in participant motion are, for whatever reason, common and inherent in MRI scanning and lead to potential artefacts and non-useable data, there is a relative lack of study into predictors of increased average motion in the first instance, particularly in generally healthy samples whose data have passed quality control (QC). This may have implications for collider bias and confounded associations.

UK Biobank is a general population cohort of 0.5 million participants who attended baseline assessment (between 2006 and 2010) and where targeted 100 000 participants will undergo MRI (including with longitudinal repeat data). We investigated associations between relatively common psychological and physical health phenotypes as well as key sociodemographic factors, with increased average motion across multiple sequences.^3^ We did not specifically hypothesize conditions that would be expected to lead to increased motion, but rather sought to determine the broad health attributes of people who had higher average motion. Currently at the time of study around 50 000 participants have completed the initial MRI scan. This study will test if increased motion (i) correlates across MRI sequences; (ii) shows longitudinal within-participant stability across time; and (iii) shows association with physical, psychological and demographic traits; and (iv) if those traits predict having ultimately complete useable data versus not.

## Materials and methods

### Study design and participants

UK Biobank is a large prospective cohort study including 502 490 general population participants who attended 1 of 22 baseline assessment centres from 2006 to 2010,^13^ aged 40–70 at baseline. In 2014, MRI scanning of the heart, brain and abdomen for a subgroup of participants began, and this is ongoing with an eventual target sample size of 100 000. As of November 2022, MRI data were available on 52 951 participants (Scan #1) who attended 4 centres (Cheadle, Newcastle, Reading and Bristol) using identical protocols. *N* = 5305 attended a second time (Scan #2; average 2.64 years later).^14^ This project was completed using UK Biobank project #17689. For each variable, we removed participants who chose not to answer/did not know; it is not clear why participants selected these options, but they were a minority of instances (<5%). We selected key non-exhaustive demographic, psychological and physical traits of interest that are commonly examined as independent variables, and based on prior work,^3^ to limit risk of Type 1 error. These were selected *a priori*, and all associations tested are reported herein.

### Imaging protocol

Image sequence, equipment, acquisition and processing details are available, open-access, on the UK Biobank website in the form of protocol (http://biobank.ctsu.ox.ac.uk/crystal/refer.cgi?id=2367) and imaging documentation (http://biobank.ctsu.ox.ac.uk/crystal/refer.cgi?id=1977). Quality controlling and additional details are described in previous open-access reports.^12,14,15^ UK Biobank provides derived IDPs that have been centrally processed and quality-controlled. This includes (i) centrally excluding scans with excessive artefacts, e.g. due to motion, and (ii) that IDPs are released to researchers corrected for motion. The outcome motion variables analysed in this report are post-QC.

### Ethical approval

This secondary-data analysis study was conducted under generic approval from the NHS National Research Ethics Service (approval letter dated 29 June 2021, Ref 21/NW/0157). Written informed consent was obtained from all participants recruited to UK Biobank.

### Demographic data

Age and sex were self-reported. Townsend deprivation index was derived from postcode of residence.^16^ This provides an area-based measure of socioeconomic deprivation derived from aggregated data on car ownership, household overcrowding, owner occupation and unemployment. Higher Townsend scores equate to higher levels of area-based socioeconomic deprivation.

### Motion data

Average head motion was indexed four ways: mean resting-state fMRI (rfMRI; https://biobank.ndph.ox.ac.uk/ukb/field.cgi?id=25741) and task-related fMRI (tfMRI; https://biobank.ndph.ox.ac.uk/ukb/field.cgi?id=25742) motion in millimetres, each averaged across space and time points, ‘measure of head motion in T_1_ structural image’ (https://biobank.ndph.ox.ac.uk/showcase/field.cgi?id=24419) and finally ‘mean absolute motion from diffusion MRI’ (https://biobank.ndph.ox.ac.uk/showcase/field.cgi?id=24450). These are described in UK Biobank imaging documentation (https://biobank.ndph.ox.ac.uk/showcase/ukb/docs/brain_mri.pdf). Results were very similar when we substituted absolute for relative measures, e.g. ‘mean relative’ versus ‘mean absolute’ motion for diffusion. We derived a ‘complete MRI data’ phenotype based on whether each participant who completed imaging had task-based fMRI data derived available for analysis^3^ (https://biobank.ndph.ox.ac.uk/showcase/field.cgi?id=25767). We used this phenotype because it was towards the end of the scan and relatively fewer participants had data here (*N* = 35 276) compared with structural or diffusion variables (e.g. arbitrarily, left amygdala grey volume *N* = 42 592; superior fronto-occipital fasciculus mean fractional anisotropy *N* = 40 318). Imaging data may not have been useable for a variety of reasons including being not completed; excess motion and/or failed registration.

Participants are instructed at the scan to keep motion to a minimum (see UK Biobank imaging documentation: https://biobank.ndph.ox.ac.uk/showcase/ukb/docs/brain_mri.pdf). The IDPs are derived centrally by UK Biobank using high-quality pipelines and shared to researchers. Where (T_1_) images are not useable for a number of reasons (e.g. missing, incomplete and gross motion) based on automated classification (where a proportion is then manually checked), the participant data are not considered further past that stage (https://biobank.ndph.ox.ac.uk/showcase/refer.cgi?id=1977). Data that are considered useable are released to researchers (such as here) and corrected by UK Biobank as standard for motion^14^ using MCFLIRT.^17^ The data and motion analysed are in participants who have passed UK Biobank QC.^12,14^ Protocol ordering was T_1_ (∼5 min), rfMRI (6 min), T_2_ FLAIR (6 min; not analysed here), diffusion tensor imaging (DTI; 7 min), susceptibility-weighted (2.5 min), Hariri faces/shapes ‘emotion’ tfMRI (4 min) and arterial spin labelling (2 min). We use motion data from T_1_, DTI, rfMRI and tfMRI data in this report.

### Blood pressure and anthropometric data

Diastolic and systolic blood pressure (DBP and SBP) were assessed using digital BP monitors (HEM-7015IT; Omron Healthcare Inc). We used the second reading because there is evidence the first reading can overestimate BP^18^ (BP were very similar if we used an average of the two). Weight was measured, to the nearest 0.1 kg, using the Tanita BC-418 MA body composition analyser. Height was measured using a Seca 202 height measure. Body mass index (BMI) was derived as weight (kg)/(height (m) × height (m) by UK Biobank centrally. Participants removed their shoes and heavy outer clothing before weight and height were measured. As a *post hoc* sensitivity analysis, in participants on antihypertensive medication, we added 5/10 mmHg to DBP/SBP, respectively, to their BP values. This is based on prior work estimating these to be approximate reductions due to medication.^19^

### Cognitive data

We examined tests that were included as part of the UK Biobank imaging cognitive assessment.^20^ One of these was a task with 13 logic/reasoning-type questions and a 2-min time limit labelled ‘fluid intelligence’ in the UK Biobank protocol and sometimes referred to as verbal–numerical reasoning. The maximum score was 13, where higher scores indicate better performance. ‘Pairs matching’ was a visuospatial memory test, where participants were asked to memorize the positions of six card pairs and then match them from memory while making as few errors as possible. Scores on this test are for the number of errors that each participant made on the single six-card round, i.e. higher scores are worse. Participants completed a timed test of symbol matching similar to the common card game Snap, which we refer to as the ‘reaction time’ (RT) task; scores are measured in milliseconds with higher values indicating worse performance.

### Psychological variables

Neuroticism was assessed using a point scale with values from 0 to 12, the Eysenck Personality Inventory Neuroticism Scale-Revised.^21-23^ Depression (yes/no for lifetime history) was based on MRI visit self-report. We derived a dichotomous variable where participants were categorized moderately/very/extremely unhappy versus moderately/very/extremely happy, based on response to the question, ‘In general, how happy are you?’.

### Lifestyle

Participants self-reported their smoking history: current, past or never. We collated past and current smokers into ‘ever’ (versus never). Participants self-reported their alcohol intake frequency on a 5-point ordinal scale from ‘never’ to ‘daily or almost daily’. We derived a dichotomous variable (‘regular drinkers’ versus not) of collated ‘daily or almost daily’, ‘three or four times a week’ and ‘once or twice a week’ versus ‘one to three times a month’, ‘special occasions only’ and ‘never’. We excluded participants from this specific variable if they reported having changed their alcohol intake due to health problems or doctor’s advice.^24^

### Physical health conditions

Using self-report, participants responded to the touch-screen question ‘Has a doctor ever told you that you have had any of the following conditions (high BP, stroke, angina and heart attack)?’. We collated heart attack and angina into coronary heart disease (CHD). Participants were also asked ‘Has a doctor ever told you that you have diabetes?’ via touch-screen.^25^ During an interview with a trained staff member, participants noted their history of any other medical conditions, and from this, we derived the phenotypes of any versus none for inflammatory conditions (e.g. rheumatoid arthritis), detailed in a previous open-access paper,^26^ and neurological conditions, listed in Supplementary Tables 1 and 2,^20^ respectively. History of traumatic brain injury was based on ICD-10 hospital records as described in a previous open-access report.^27^ Participants self-reported regular medication for cholesterol, BP, diabetes and/or taking exogenous hormones, from which we derived a dichotomous variable (any versus none). Participants self-reported their overall health as excellent, good, fair or poor. We derived a dummy variable: excellent/good versus fair (i.e. good health).

### Statistical analyses

We used Pearson *r* correlations for intercorrelations between the multiple head motion phenotypes. Linear regressions were used to estimate associations between each of the multiple phenotypes versus each of the dependent variables of rfMRI and structural MRI motion. Associations were standardized (other than where stated otherwise) such that a beta = 0.5 reflects a 0.5 standard deviation (SD) difference in motion per 1 SD increase in the predictor, as well as 95% confidence intervals (CIs) and uncorrected *P*-values. We controlled for age and sex in association analyses. To limit Type 1 error, we conservatively set statistical significance at *P* ≤ 0.001. While the *a priori* analysis plan included prediction of motion and complete data versus not, certain *post hoc* analyses were performed (e.g. adjusting BP measurements for self-reported medication^19^), and these are described under ‘Sensitivity analyses’.

## Results

A total of *N* = 52 951 participants attended (Scan #1; mean age = 64.48, SD = 7.81), and *N* = 5305 had attended the repeat visit (mean age = 64.46 at first scan, SD = 7.30; mean interval 2.64 years). Of the people who attended Scan #1, *n* = 25 578 (48%) were male. All motion variables, RT and pair-matching errors had non-normal distributions and were transformed with a natural-log function.

All fMRI (log-)motion variables—first and repeat scans for rfMRI and tfMRI—were strongly intercorrelated (around Pearson *r* = 0.7–0.8, *P* < 0.001), with weaker correlations for structural T_1_ motion (around *r* = 0.3), and weaker again for diffusion (around *r* = 0.1) as shown in Table 1. From this point, we do not present tfMRI further as results were very similar to fMRI, and given the aforementioned correlations. *T*-tests showed significant differences in motion by ‘MRI data complete?’ status (rfMRI *t* = 6.87, *P* < 0.001; Cohen’s *D* = 0.1; structural MRI *t* = 19.08, *P* < 0.001; Cohen’s *D* = 0.2) where people with complete data had less motion. By contrast, participants with complete data had higher diffusion motion on average (*t* = 6.87, *P* < 0.001; Cohen’s *D* = 0.2).

**Table 1:** Stability of motion variables across variables and scan visit

	rfMRI first scan (95% CIs)	rfMRI second scan (95% CIs)	tfMRI first scan (95% CIs)	tfMRI second scan (95% CIs)	Structural MRI first scan (95% CIs)	Structural MRI second scan (95% CIs)	Diffusion MRI first scan (95% CIs)	Diffusion MRI second scan (95% CIs)
rfMRI first scan (95% CIs)	1							
rfMRI second scan (95% CIs)	0.771*** (0.759; 0.783)	1						
tfMRI first scan (95% CIs)	0.796*** (0.793; 0.800)	0.677*** (0.660; 0.693)	1					
tfMRI second scan (95% CIs)	0.673*** (0.656; 0.690)	0.778*** (0.766; 0.790)	0.724*** (0.708; 0.739)	1				
Structural MRI first scan (95% CIs)	0.248*** (0.239; 0.257)	0.183*** (0.155; 0.211)	0.196*** (0.186; 0.206)	0.161*** (0.131; 0.190)	1			
Structural MRI second scan (95% CIs)	0.249*** (0.222; 0.275)	0.287*** (0.260; 0.313)	0.219*** (0.191; 0.246)	0.226*** (0.196; 0.254)	0.382*** (0.358; 0.405)	1		
Diffusion MRI first scan (95% CIs)	0.0939*** (0.084; 0.104)	0.0499*** (0.021; 0.079)	0.0909*** (0.081; 0.101)	0.0595*** (0.029; 0.090)	0.00185 (−0.007; 0.011)	−0.00562 (−0.033; 0.022)	1	
Diffusion MRI second scan (95% CIs)	0.0346* (0.007; 0.063)	0.0956*** (0.067; 0.124)	0.0341* (0.005; 0.063)	0.0777*** (0.047; 0.108)	−0.000561 (−0.028; 0.027)	0.0569*** (0.030; 0.084)	0.162*** (0.136; 0.189)	1

*P < 0.05, **P < 0.01, ***P < 0.001. Notes: Pearson intercorrelations between log-rfMRI, log-tfMRI, T_1_ structural and diffusion imaging head motion first and second scans. CI, confidence interval.

### Sex and age trajectories

For fMRI, there were significant sex differences in (raw) motion (higher in males versus female β = 0.281, 95% CI = 0.262–0.300, *P* < 0.001), and with increasing age (β = 0.025 per year, 95% CI = 0.023–0.026, *P* < 0.001). There was evidence of a sex/age interaction (β = 0.029 for females, β = 0.017 for males; interaction *P* < 0.001), reflecting a steeper age effect—from a lower baseline—in females. There was no evidence of a non-linear age effect.

**Figure 1:**
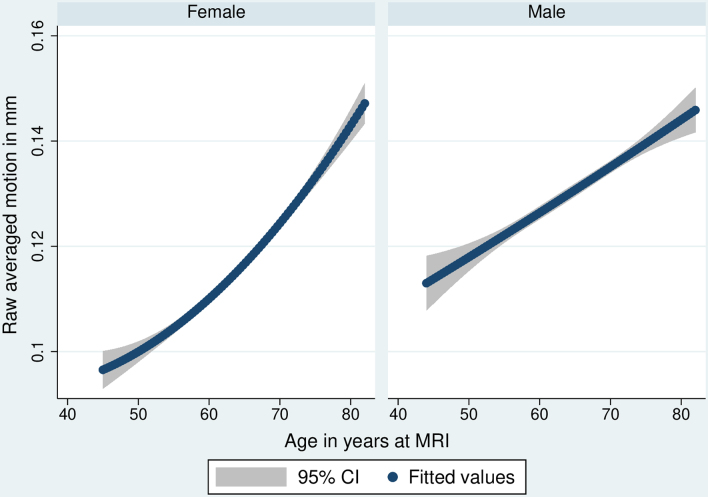
**Sex-specific cross-sectional age trajectories of rfMRI motion, averaged across space and time points.** Quadratic linear regressions showing average trajectories, including 95% CI. Standardized β = 0.029 per year for females, β = 0.017 for males; both *P* < 0.001.

**Figure 2:**
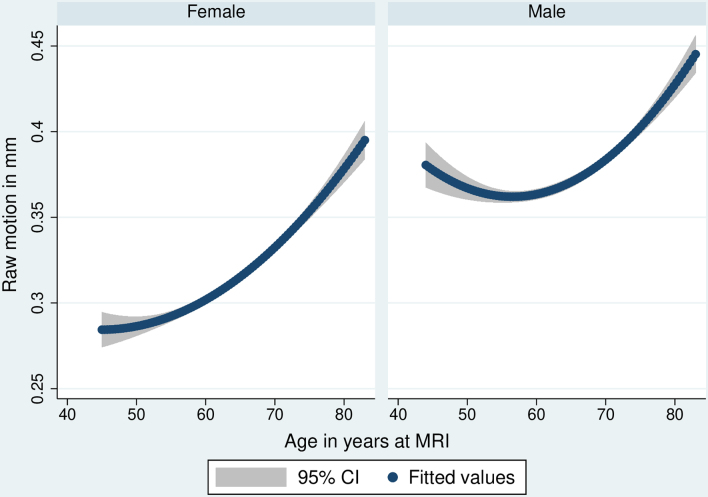
**Sex-specific cross-sectional age trajectories of T_1_ structural MRI motion.** Quadratic linear regressions showing average trajectories, including 95% CI. Standardized β = 0.020 per year for females, β = 0.010 for males, both *P* < 0.001.

Findings were similar for structural T_1_ motion including an overall sex effect (higher in males; β = 0.390, 95% CIs 0.373–0.407, *P* < 0.001). There were sex-specific age trajectories (β = 0.020 per year for females, β = 0.010 for males, each *P* < 0.001; interaction *P* < 0.001). There was evidence of a non-linear age effect (*P* < 0.001). When stratified by a median split (≥65 versus < 65 years), there was evidence of further sex interaction (*P* < 0.001). Below 65, increasing age associated with higher motion in females only (β = 0.018, *P* < 0.001; male *P* = 0.226). At 65 and above, the slope was steeper for females versus males (female β = 0.038, male β = 0.025, both *P* < 0.001). Functional and structural T_1_ trajectories are shown in Figs. 1 and 2.

For diffusion motion, there was evidence of a sex effect (lower overall in males: β = −0.112, 95% CI = −0.130 to −0.094, *P* < 0.001). A linear age effect was null (*P* = 0.117), where adding a quadratic term (i.e. age^2^) showed a non-linear age effect (age β = 0.068, age^2^ β = −0.005, both *P* < 0.001). There were no sex interactions in this context. Stratified by median split, there was a positive association between increasing age and diffusion motion until 65 years (β = 0.005, 95% CIs = 0.002 to 0.008, *P* < 0.001), and a negative association thereafter (β = −0.011, 95% CIs = −0.014 to −0.007, *P* < 0.001). These non-linear effects are displayed in Supplementary Fig. 1. When we added a quadratic age term to subsequent association analyses, this made no difference to any final results. Scatterplots for all age/sex/motion variables are displayed in Supplementary Figs. 2–4.

### Associations between individual predictors and motion

Participants with incomplete (fMRI) data (*N* = 5522) were excluded for tests of association between predictors and motion. The majority of predictor variables were associated with rfMRI motion in the expected directions (e.g. higher BMI, history of CHD and worse memory with increased motion; see Table 2). The effect sizes varied from standardized β = 0.053 for log RT to β = 0.664 for diabetes (all *P* ≤ 0.033). Regular drinking (versus not) was associated with lower average motion (beta = −0.151, *P* < 0.001). The only exceptions (i.e. non-significant) were log pair-matching errors and history of traumatic brain injury, which did not show evidence of a statistically significant association (*P* > 0.100).

**Table 2:** Associations between psychological and physical health, with average log resting-state motion

	Outcome	β	SE	*P*-value	CI (lower)	CI (upper)	*N*	*r* ^2^
Sociodemographic	Townsend deprivation index	**0.076**	**0.006**	**<0.001**	**0.064**	**0.087**	**35 207**	**0.052**
	Regular drinker (versus not)	**−0.151**	**0.015**	**<0.001**	**−0.181**	**−0.122**	**33 884**	**0.054**
	Ever-smoker (versus never)	**0.188**	**0.011**	**<0.001**	**0.167**	**0.209**	**34 916**	**0.059**
Psychological health	Good health (versus not)	**−0.473**	**0.014**	**<0.001**	**−0.500**	**−0.446**	**34 342**	**0.084**
	Happy (versus not)	**0.059**	**0.028**	**0.033**	**0.005**	**0.113**	**34 922**	**0.050**
	Depression (versus not)	**0.191**	**0.021**	**<0.001**	**0.150**	**0.232**	**35 240**	**0.052**
	Neuroticism	**0.020**	**0.005**	**<0.001**	**0.010**	**0.030**	**35 033**	**0.050**
	Log pair-matching errors	0.001	0.005	0.914	−0.010	0.011	33 221	0.050
	Log reaction time	**0.053**	**0.006**	**<0.001**	**0.042**	**0.064**	**33 023**	**0.052**
	Fluid intelligence	**−0.091**	**0.005**	**<0.001**	**−0.101**	**−0.081**	**32 585**	**0.058**
Physical health								
	BMI	**0.619**	**0.004**	**<0.001**	**0.611**	**0.628**	**33 988**	**0.427**
	SBP	**0.160**	**0.006**	**<0.001**	**0.148**	**0.172**	**28 216**	**0.074**
	DBP	**0.195**	**0.006**	**<0.001**	**0.184**	**0.207**	**28 219**	**0.089**
	Cardiovascular medication (versus not)	**0.324**	**0.015**	**<0.001**	**0.294**	**0.354**	**35 166**	**0.062**
	Neurological condition (versus not)	**0.066**	**0.031**	**0.034**	**0.005**	**0.127**	**35 240**	**0.050**
	Inflammatory condition (versus not)	**0.247**	**0.013**	**<0.001**	**0.221**	**0.272**	**33 736**	**0.060**
	CHD (versus not)	**0.393**	**0.053**	**<0.001**	**0.289**	**0.496**	**35 227**	**0.051**
	High BP (versus not)	**0.380**	**0.020**	**<0.001**	**0.341**	**0.419**	**35 227**	**0.059**
	Diabetes (versus not)	**0.664**	**0.023**	**<0.001**	**0.619**	**0.710**	**34 931**	**0.072**
	Traumatic brain injury (versus not)	0.056	0.074	0.447	−0.088	0.200	35 240	0.050

Note: linear regression associations are standardized on the per-SD scale (e.g. β = 0.02 SD increased motion per SD of neuroticism score). Bold type indicates statistically significant results. Scores are in people with ‘useable’ data only. SE, standard error; CI, confidence interval.

In terms of structural T_1_ motion, findings were similar but generally had smaller effect sizes (see Table 3). The depression association with rfMRI flipped sign to become slightly positive/protective (i.e. people with depression had less structural MRI motion; β = −0.077, *P* < 0.001), higher pair-matching (memory) errors became significantly associated with increased motion (β = 0.018, *P* < 0.001), the BMI association attenuated substantially (from β = 0.6 to β = 0.119, *P* < 0.001), as did the diabetes association (β = 0.664 to β = 0.173, *P* < 0.001), and CHD and high BP flipped sign to become positive (i.e. lower motion; CHD β = −0.476, *P* < 0.001; high BP β = −0.503, *P* < 0.001).

**Table 3:** Associations between psychological and physical health, with average structural motion

	Outcome	β	SE	*P*-value	CI (lower)	CI (upper)	*N*	*r* ^2^
Sociodemographic	Townsend deprivation index	**0.051**	**0.006**	**<0.001**	**0.040**	**0.063**	**35 174**	**0.045**
	Regular drinker (versus not)	0.009	0.016	0.564	−0.022	0.040	33 855	0.043
	Ever-smoker (versus never)	**0.063**	**0.011**	**<0.001**	**0.042**	**0.084**	**34 882**	**0.044**
Psychological health	Good health (versus not)	**−0.082**	**0.015**	**<0.001**	**−0.111**	**−0.054**	**34 309**	**0.044**
	Happy (versus not)	0.040	0.029	0.161	−0.016	0.096	34 889	0.043
	Depression (versus not)	**−0.077**	**0.022**	**<0.001**	**−0.119**	**−0.035**	**35 207**	**0.043**
	Neuroticism	−0.002	0.005	0.701	−0.013	0.009	34 999	0.043
	Log pair-matching errors	**0.018**	**0.005**	**0.001**	**0.007**	**0.029**	**33 189**	**0.043**
	Log reaction time	**0.034**	**0.006**	**<0.001**	**0.022**	**0.045**	**32 991**	**0.043**
	Fluid intelligence	**−0.032**	**0.005**	**<0.001**	**−0.043**	**−0.021**	**32 555**	**0.045**
Physical health								
	BMI	**0.119**	**0.006**	**<0.001**	**0.108**	**0.130**	**33 957**	**0.056**
	SBP	**0.059**	**0.006**	**<0.001**	**0.046**	**0.071**	**28 176**	**0.047**
	DBP	**0.023**	**0.006**	**<0.001**	**0.011**	**0.035**	**28 179**	**0.045**
	Cardiovascular medication (versus not)	**0.037**	**0.016**	**0.019**	**0.006**	**0.068**	**35 133**	**0.043**
	Neurological condition (versus not)	0.003	0.032	0.914	−0.059	0.066	35 207	0.043
	Inflammatory condition (versus not)	<0.001	0.013	0.986	−0.027	0.026	33 701	0.042
	CHD (versus not)	**−0.476**	**0.055**	**<0.001**	**−0.583**	**−0.369**	**35 194**	**0.045**
	High BP (versus not)	**−0.503**	**0.021**	**<0.001**	**−0.544**	**−0.463**	**35 194**	**0.059**
	Diabetes (versus not)	**0.173**	**0.024**	**<0.001**	**0.126**	**0.221**	**34 898**	**0.044**
	Traumatic brain injury (versus not)	−0.079	0.075	0.298	−0.227	0.069	35 207	0.043

Note: linear regression associations are standardized on the per-SD scale (e.g. β = 0.02 SD increased motion per SD of neuroticism score). Bold type indicates statistically significant results. Scores are in people with ‘useable’ data only. SE, standard error; CI, confidence interval.

Results for motion during diffusion imaging showed generally fewer associations (Supplementary Table 3): 8 of 20 tests of association were significant at *P* < 0.05 compared with the majority for fMRI/structural motion. History of depression, more log pair-matching errors, worse fluid intelligence scores, inflammatory conditions and diabetes were associated with increased motion (β range [−]0.015 to 0.071, highest *P* = 0.027). Some associations were in unexpected positive directions: regular drinking and raised SBP/DBP were associated with less motion (β range = −0.015 to −0.04, highest *P* = 0.014).

### Associations between individual predictors and complete data

We tested for sociodemographic and health differences in participants with versus without useable, complete MRI data. Generally worse values (psychological, physical and demographic) were associated with significantly lower odds of complete data (Table 4). To reduce the possibility that some in the ‘incomplete data’ category were such because their IDP data was simply not processed/available yet, we excluded participants with a first scan data after May 2021 (*n* = 3919 or 7.4%; noting that scanning stopped from ∼Q1 2020 to Q3 2021 due to COVID-19). Results were very similar with no meaningful differences.

**Table 4:** Associations between psychological and physical health, with having complete useable data versus not

	Outcome	OR	SE	*P*-value	CI (lower)	CI (upper)	*N*	*r* ^2^
Sociodemographic	Age (per year)	**0.964**	**0.001**	**<0.001**	**0.962**	**0.967**	**50 376**	**0.014**
	Sex (male)	**0.908**	**0.018**	**<0.001**	**0.873**	**0.944**	**50 376**	**0.014**
	Townsend deprivation index	**0.924**	**0.010**	**<0.001**	**0.905**	**0.944**	**50 326**	**0.015**
	Regular drinker (versus not)	**1.196**	**0.034**	**<0.001**	**1.132**	**1.265**	**48 282**	**0.015**
	Ever-smoker (versus never)	0.990	0.020	0.627	0.951	1.031	49 824	0.014
Psychological health								
	Good health (versus not)	**1.302**	**0.034**	**<0.001**	**1.237**	**1.370**	**48 848**	**0.016**
	Happy (versus not)	**0.891**	**0.048**	**0.030**	**0.802**	**0.989**	**49 844**	**0.014**
	Depression (versus not)	0.970	0.040	0.468	0.896	1.052	50 376	0.014
	Neuroticism	0.985	0.010	0.132	0.866	1.005	49 994	0.014
	Log pair-matching errors	**0.970**	**0.010**	**0.003**	**0.950**	**0.990**	**47 121**	**0.013**
	Log reaction time	**0.960**	**0.010**	**<0.001**	**0.940**	**0.980**	**46 807**	**0.013**
	Fluid intelligence	**1.095**	**0.011**	**<0.001**	**1.073**	**1.117**	**46 233**	**0.014**
Physical health								
	BMI	**0.897**	**0.009**	**<0.001**	**0.870**	**0.915**	**48 407**	**0.016**
	SBP	**0.909**	**0.011**	**<0.001**	**0.889**	**0.931**	**39 820**	**0.015**
	DBP	**0.943**	**0.011**	**<0.001**	**0.922**	**0.964**	**39 824**	**0.015**
	Cardiovascular medication (versus not)	**0.917**	**0.027**	**0.003**	**0.866**	**0.971**	**50 260**	**0.014**
	Neurological condition (versus not)	**0.769**	**0.041**	**<0.001**	**0.692**	**0.855**	**50 376**	**0.015**
	Inflammatory condition (versus not)	**0.897**	**0.022**	**<0.001**	**0.854**	**0.941**	**48 090**	**0.015**
	CHD (versus not)	**1.524**	**0.171**	**<0.001**	**1.223**	**1.898**	**50 357**	**0.015**
	High BP (versus not)	**1.807**	**0.084**	**<0.001**	**1.649**	**1.981**	**50 357**	**0.017**
	Diabetes (versus not)	**0.756**	**0.031**	**<0.001**	**0.698**	**0.818**	**49 844**	**0.015**
	Traumatic brain injury (versus not)	**0.720**	**0.091**	**0.009**	**0.562**	**0.923**	**50 376**	**0.014**

Note: all continuous predictors are standardized on the per-SD scale (e.g. OR = 0.897 of complete useable data per SD higher BMI). Bold type indicates statistically significant logistic regression results. OR, odds ratio; SE, standard error; CI, confidence interval.

### Sensitivity analyses

BP results were unchanged when we added 5/10 mmHg to DBP/SBP, respectively, for participant imaging values if they also reported antihypertensive medication. We re-ran predictor/motion association tests having removed participants with a self-reported neurological condition (*n* = 1605 or 3%) that may lead to tremors and involuntary movements, such as Parkinson’s disease (Supplementary Table 2). Those results were very similar (Supplementary Tables 4 and 5). Results were fundamentally very similar when we performed unadjusted regressions, i.e. did not control for age and sex (Supplementary Tables 6 and 7), with betas mostly changing at the first or second decimal point. Certain associations were significant at nominal *P* < 0.05 but not at the more conservative *P* ≤ 0.001: happy versus not; neurological conditions versus not with average rfMRI motion; cardiovascular medication versus not and structural motion; and happy versus not; pair-matching errors; cardiovascular medication versus not; traumatic brain injury versus not; and having complete data (versus not).

## Discussion

Prior research suggests that participant motion in MRI is a substantial and common confounder in understanding brain structure and function. Motion increases the risk of image artefacts and, beyond certain levels, risk of exclusion from the study completely. Here, in ∼50 000 generally healthy participants who passed QC, we demonstrate that increased participant motion during fMRI and structural MRI scanning (largely) positively correlates longitudinally across time and scanner sequences and can be somewhat predicted by poorer sociodemographic, psychological and physical health attributes (with specific exceptions). Poorer predictor values were generally associated with significantly lower odds of ‘complete/useable’ data. Importantly, associations between poorer health and generally increased motion remained even when we restricted analyses to participants with useable data.

There are implications to this report’s findings. UK Biobank is known for pronounced healthy participation bias generally.^28^ We have previously shown that poorer health is a risk factor for non-participation bias (in UK Biobank imaging)^3^—here, we extend that to demonstrate biases even within the participant sample who did attend imaging. Healthier participants move less, suggesting a bias towards better health in people whose images are at potentially lower risk of artefacts and exclusion. This has possible implications for the field in terms of understanding brain substrates of psychological behaviour and neuropsychiatric/degenerative conditions, as well as diagnostic use and risk prediction. It should be noted however that beyond scans excluded at the QC stage, UK Biobank IDPs are post-correction (including for measured motion), and therefore, it is not necessarily the case that these biases substantially affect the image quality in released, widely used data. These findings do however reinforce the need for explicit reporting of confound-related QC values in study reports, including motion.^8^

There were some unexpected findings. Regular drinking was associated with less (fMRI) motion, having excluded participants who do not drink for health reasons and/or neurological conditions. It has been observed before that relatively frequent non-problematic drinking can be a (non-causal) marker of good health^29^; this is a potential explanation. There were a few associations in unexpected directions (i.e. poorer health and less motion) that differed by sequence e.g. between high BP and CHD with less structural T_1_ motion and with better odds of useable data (among other examples), and it is not clear why. We observed only a moderate significant association between self-reported neurological conditions (e.g. Parkinson’s disease) and (fMRI) motion. This was expected due to common symptoms like tremors and/or cognitive problems (where cognitive scores did show association). It is possible that participants with severe difficulty lying still either did not attend assessment, possessed contraindications prohibiting scan or moved to such a degree they were excluded prior to processing.^12^ This is supported by the observation that participants with neurological conditions had substantially increased risk of incomplete data.

We report a number of associations between health conditions without obvious (causal) effects on increased motion, e.g. CHD. These may reflect a very generalized, non-specific association between poorer health and increased motion. The ordering of scan sequences was T_1_, rfMRI, diffusion and then tfMRI. It is possible that the occasional differences in results between sequences are due to increased tiredness across the protocol, where T_1_ was first and participants may be expected to move the least. While most associations were strongly statistically significant at *P* < 0.001, a number were only marginally significant (*P* < 0.05) and therefore not so at more conservative thresholds. We elected for *P* < 0.001 as an approximate more robust level of significance, where more conservative thresholds may result in some associations becoming non-significant. This report includes several hundred (correlated) individual tests of association, of which a significant number are *post hoc* sensitivity checks (e.g. changes to exclusion criteria). In that context, we encourage interpretation less on formal statistical significance but rather the magnitude of effect size—most of which are generally small.

We showed substantial cross-sequence and cross-visit correlations between functional and T_1_ structural motion variables. If a participant has shown substantial movement artefacts, additional physical support measures may be required to reduce movement subsequently. Quantification of movement and incorporation into ‘best practice’ could be considered in future studies. There is evidence that participation/healthy volunteer bias has significant influence over effect estimates in UK Biobank; explicit correction for the underrepresentation of relevant data (here imaging) in people with poorer health may be an important avenue of study.^30^ On the basis of these findings, we have identified easily measured and common potential risk factors for increased motion before participants commence scanning.

### Limitations

UK Biobank has known healthy participant bias,^28^ and more so for the imaging subsample.^3^ This could lead to underestimates of correlations between poorer health and motion, because participants with particularly poor health may be unable to attend or complete the scan.

Increased motion was a risk factor for not having useable (available/complete) data based on the task fMRI IDP, although this could be for a variety of reasons not necessarily specific to motion, and including that the participant image has simply not been processed as of yet. That said, the majority of the ∼50k scans were prior to April 2020, when COVID-19 necessitated stopping scanning for a prolonged period. This means that the effect is unlikely to be due to those participants not having derived data as of early 2023, especially given the findings would necessitate a non-random reason why participants who moved more had delayed imaging processing.

The phenotypes examined (deprivation, physical health, etc.) were selected *a priori* because they are relatively common variables in epidemiological/UK Biobank analyses. These are however non-exhaustive and somewhat broad (e.g. depression history overall rather than more fine-grained metrics like age at onset). Future study will investigate the role of multiple neuropsychiatric phenotypes (including subdivisions) on motion and data completeness.

We based complete data versus not on fMRI because it had relatively high incompleteness compared with structural/diffusion IDPs. There are other ways researchers could derive a complete versus incomplete data phenotype, including having missing data on any IDP (where there are several hundred).

While the motion metrics were strongly intercorrelated across rfMRI/tfMRI, these values were averaged across the scan duration and may not perfectly characterize motion for all people. Some participants may have moved infrequently but to a large degree while others more subtly but frequently, and this may affect parts of the sequence differently. The different motion metrics were slightly distinct, e.g. fMRI averaged across time/space versus absolute diffusion motion.

While motion is an important factor, there are others such as scanner table position that play a role in confounding exposure/outcome associations.^12^ There are likely some sequence-specific variations in what contributes to motion/artefacts, e.g. vulnerability to cardiac cycles.^31^ This may contribute to relatively inconsistent correlations where the two fMRI modalities (resting-state; task) correlated strongly, less so with T_1_ structural, and even less so with diffusion (and where the latter two correlated nominally significantly but relatively weakly). The current findings use released IDPs subsequent to (central UK Biobank) motion correction. This is distinct from scans excluded at an early, preprocessing stage.^12^The amount of motion reported here is probably to some extent underestimated because participants with clearly unusable T_1_ scan data (e.g. due to severe motion or artefacts) were previously removed.^12^

### Future research

This report examined around 50 000 participants who attended imaging and around 5000 with repeat data. This will increase to a target of *n* = 100 000 including 60 000 between 2 and 7 years apart (https://www.ukbiobank.ac.uk/learn-more-about-uk-biobank/news/ambitious-project-announced-to-create-the-world-s-largest-longitudinal-imaging-dataset). Replicating these results in the larger, full cohort will be valuable. Future research may investigate broader demographic biases, e.g. ethnicity and deprivation, and/or whether the examined predictors associate with more or less stability in motion across time. Neuroimaging is a common approach to understanding neuropsychiatric phenotypes (e.g. schizophrenia, depression), and more detailed consideration of such variables would be informative. This could include depression sub-phenotypes, and self-reported versus ICD-9/10–coded instances. Imaging is confounded by a range of variables, each of which has a variety of correction approaches.^9^ Future studies should additionally investigate a wider range of confounds in tandem with average motion, as well as applying finer-grained measures to the type and frequency of motion, in correlating with psychological and physical health. Different motion correction techniques may result in different observations.^32^ We used four measures of motion whereas a wide number of more discrete measurements exist that could be examined.^12^

The effect sizes of predictors versus motion were often relatively small particularly for T_1_ structural and DT MRI. It should be noted that IDPs released to researchers by UK Biobank are subsequent to correction for motion—we do not provide evidence here regarding if the associations specifically influence other exposure/outcome correlations. While significant motion may suggest benefit for physical support measures in at-risk populations, the benefit of that would need to be demonstrated empirically.

## Conclusion

Head motion during MRI scanning is a confound to understanding brain structure and function.^12^ It can lead to non-useable/incomplete images and/or artefacts that can influence the quality and accuracy of data. We have previously demonstrated small but prevalent ‘participation bias’ in UK Biobank imaging compared with baseline participants, themselves a relatively biased subsample of the UK population. In this report, we show that increased motion is (i) a relatively stable trait across scan sequences and visits; (ii) associated with a wide range of poorer psychological and physical health attributes; and (iii) in the case of fMRI and T_1_ structural imaging motion, associated with lower likelihood of having useable data. These findings have implications for representativeness within population health sciences. There is evident bias towards healthier participants attending imaging but also in their likelihood of minimizing average motion and, separately, having useable data.

## Supplementary Material

fcae220_Supplementary_Data

## Data Availability

UK Biobank is an open-access resource available to verified researchers upon application (http://www.ukbiobank.ac.uk/). Analysis syntax is available from the authors directly upon request.
