# Supplementary material for: Head motion in the UK Biobank imaging subsample: longitudinal stability, associations with psychological and physical health, and risk of incomplete data
Source: Brain Commun. 2024 Jul 2;6(4):fcae220. doi: 10.1093/braincomms/fcae220 (PMC11249925; doi:10.1093/braincomms/fcae220)
Supplement: fcae220_Supplementary_Data [file fcae220_Supplementary_Data.zip › Supplementary Figures 1-4.pdf]

Supplementary Figure 1: sex-specific cross-sectional age trajectories of diffusion MRI motion.

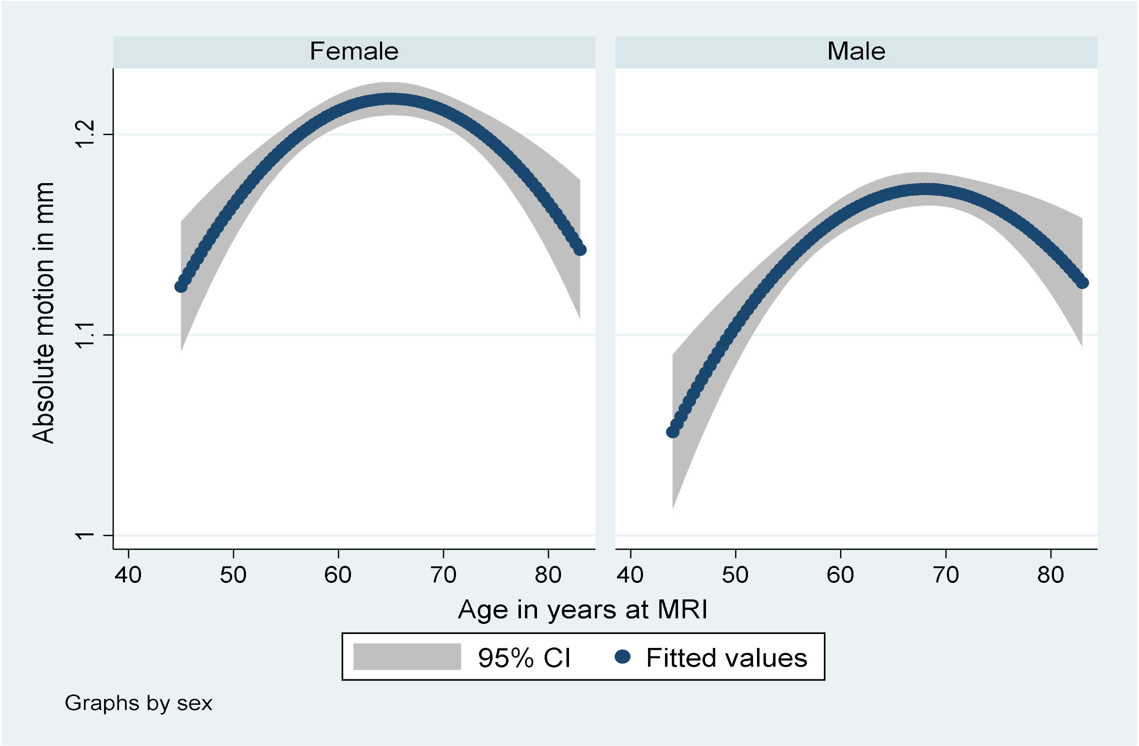

Supplementary Figure 2: raw motion/age scatterplots (sex-stratified) for resting-state functional magnetic resonance imaging (rfMRI), including quadratic estimates and 95% confidence intervals (CIs)

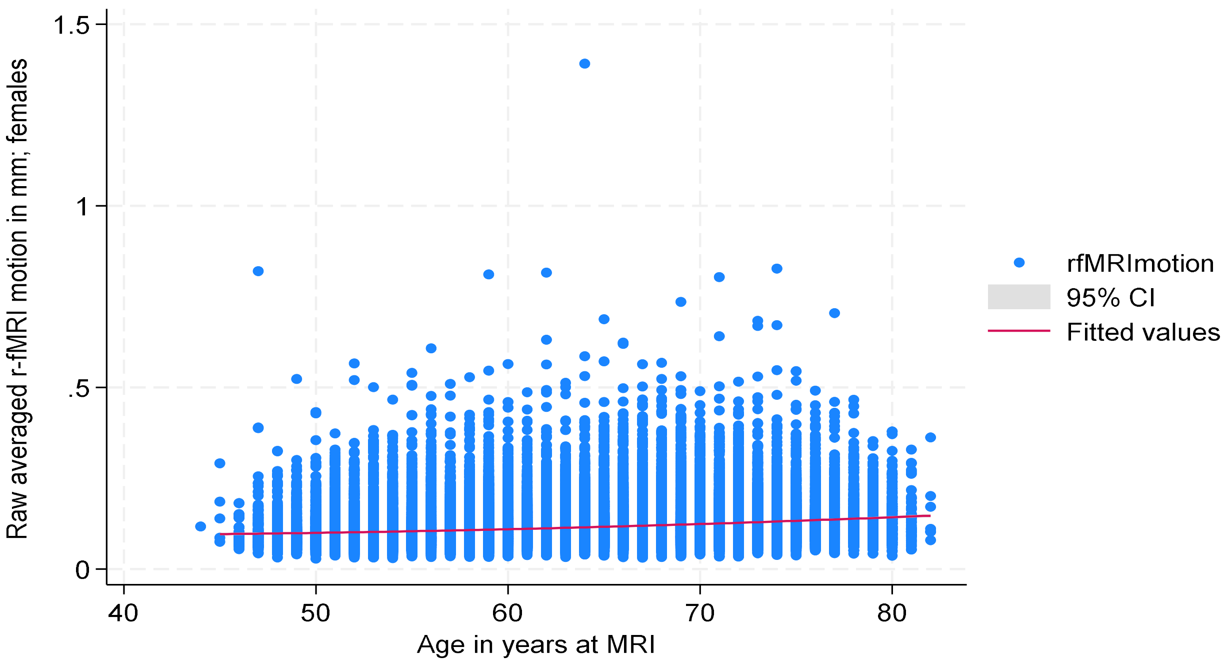

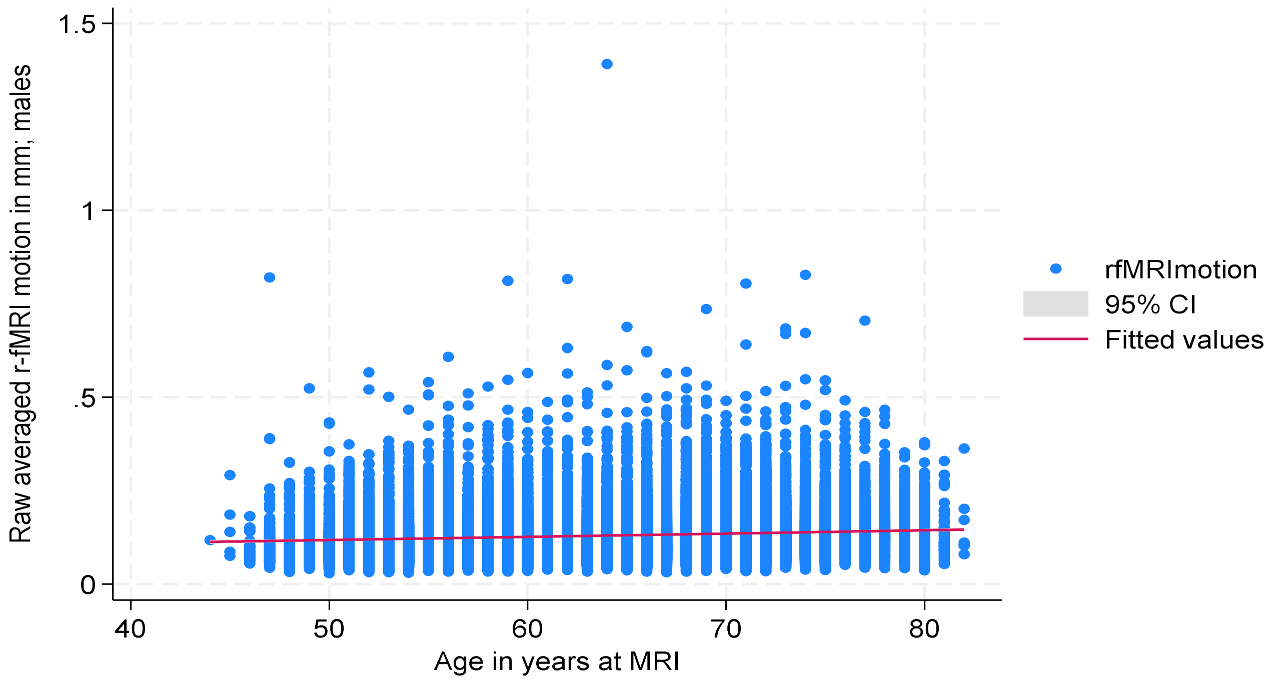

Note: metrics are described in detail in UK Biobank imaging documentation at: https://biobank.ndph.ox.ac.uk/showcase/ukb/docs/brain_mri.pdf

Supplementary Figure 3: raw motion/age scatterplots (sex-stratified) for structural motion (StMotion), including quadratic estimates and 95% confidence intervals (CIs)

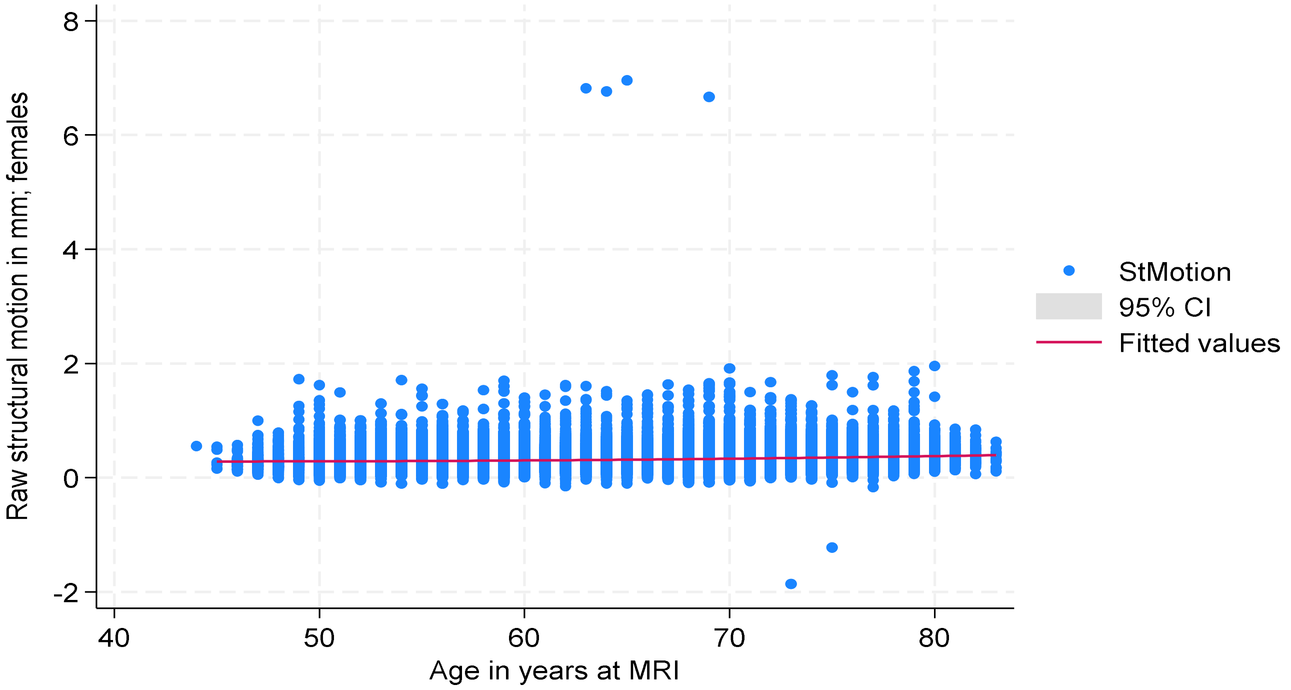

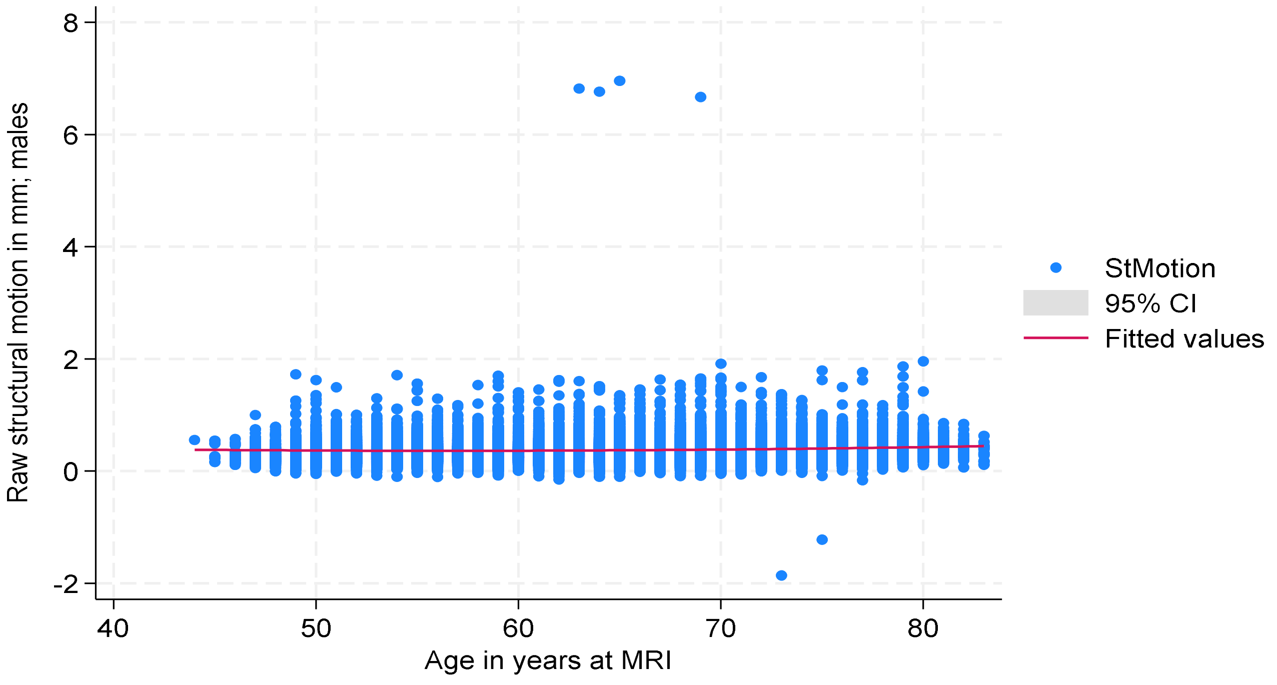

Note: metrics are described in detail in UK Biobank imaging documentation at:
https://biobank.ndph.ox.ac.uk/showcase/ukb/docs/brain_mri.pdf

Supplementary Figure 4: raw motion/age scatterplots (sex-stratified) for diffusion tensor imaging motion (DiMotion) including quadratic estimates and 95% confidence intervals (CIs)

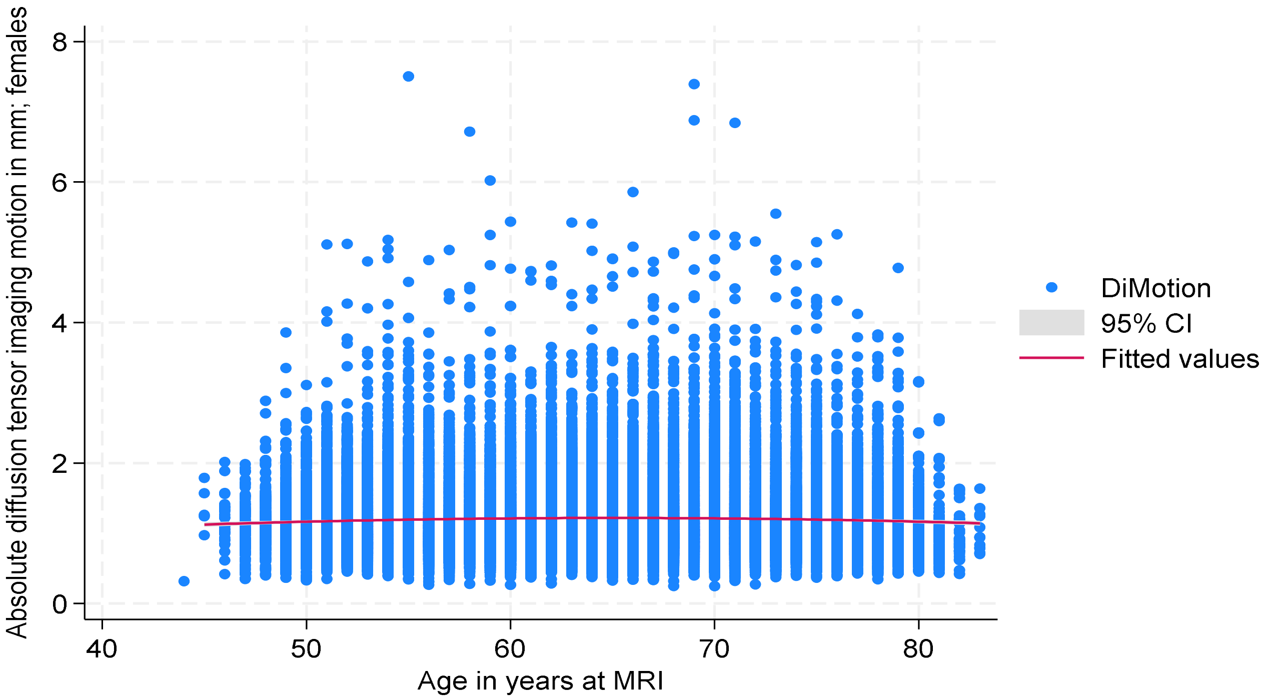

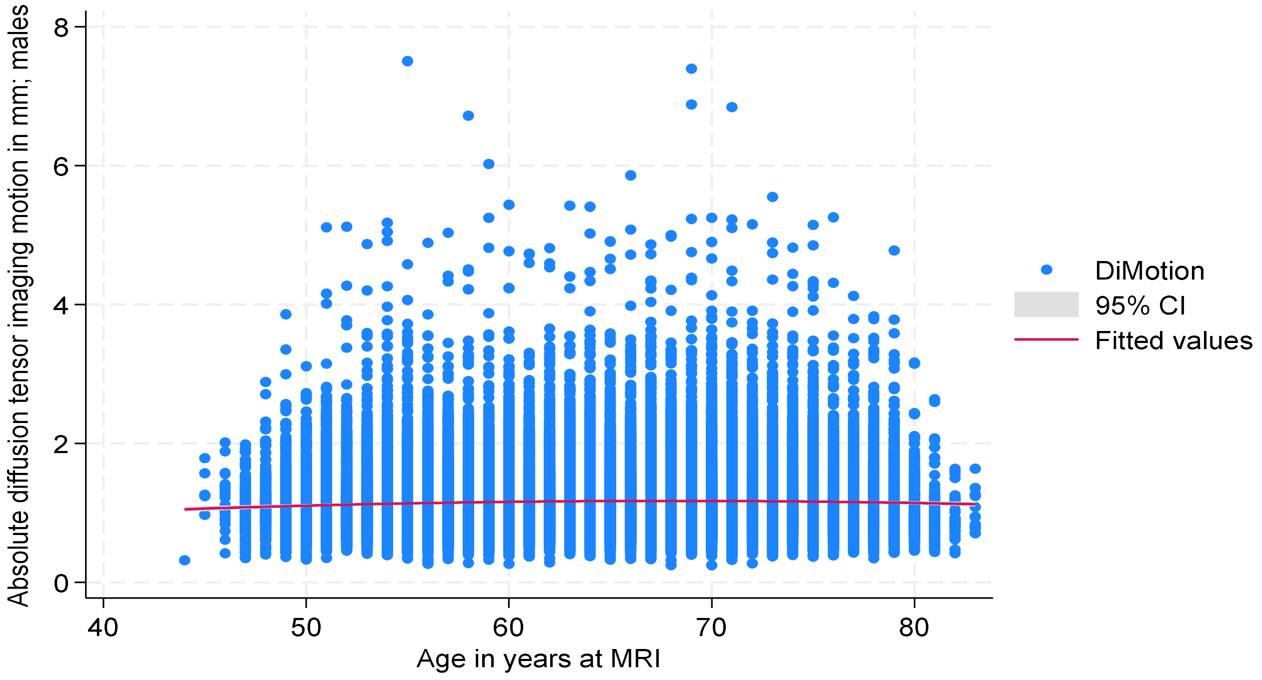

Note: metrics are described in detail in UK Biobank imaging documentation at: https://biobank.ndph.ox.ac.uk/showcase/ukb/docs/brain_mri.pdf
